# The Predictive Role of Tolerance and Health Problems in Problem Gambling: A Cross-Sectional and Cross-Lagged Network Analyses

**DOI:** 10.1007/s10899-023-10191-5

**Published:** 2023-02-04

**Authors:** Zsolt Horváth, Borbála Paksi, Fernando Fernández-Aranda, Susana Jiménez-Murcia, Zsolt Demetrovics

**Affiliations:** 1 Institute of Psychology, ELTE Eötvös Loránd University, Izabella Utca 46, Budapest, 1064 Hungary; 2 Centre of Excellence in Responsible Gaming, University of Gibraltar, Gibraltar, Gibraltar; 3 Institute of Education, ELTE Eötvös Loránd University, Budapest, Hungary; 4 Department of Psychiatry, University Hospital of Bellvitge, Barcelona, Spain; 5 CIBER Fisiopatología Obesidad y Nutrición (CIBERObn), Instituto de Salud Carlos III., Madrid, Spain; 6 Psychoneurobiology of Eating and Addictive Behaviors Group, Neurosciences Programme, Bellvitge Biomedical Research Institute (IDIBELL), Barcelona, Spain; 7 Department of Clinical Sciences, School of Medicine and Health Sciences, University of Barcelona, Barcelona, Spain

**Keywords:** Cross-lagged panel network model, Gambling disorder, Harmful gambling, Network analysis

## Abstract

The existing symptomatic networks of problem gambling are all based on cross-sectional data. Thus, there is a need to explore longitudinal symptom networks of problem gambling. Moreover, the replicability of cross-sectional symptom networks can be limited; therefore, further research should assess the convergence between cross-sectional networks of problem gambling symptoms. The present study aimed (i) to examine cross-sectional networks of problem gambling symptoms and evaluate their replicability and (ii) to examine a longitudinal cross-lagged network of problem gambling symptoms. The study included a representative sample of young adult gamblers (born between 1984 and 2000) from the first two waves of the Budapest Longitudinal Study (original sample: *N* = 2777; final sample: *N* = 335). The Problem Gambling Severity Index was used to assess symptoms of problem gambling. Cross-sectional symptom networks showed differences in the centrality of nodes. Correlations between the two cross-sectional networks were low in the presence vs. absence of edges, rank order of edge weights, and centrality estimates. However, network invariance tests indicated non-significant differences between them. The cross-lagged network revealed that the symptoms of tolerance and health problems could predict the subsequent presence of multiple problem gambling symptoms. Overall, limited evidence demonstrated the replicability of cross-sectional symptom networks of problem gambling. Future research needs to explore the utility of cross-sectional networks of problem gambling and assess more precisely causal relationships between problem gambling symptoms by distinguishing within- and between-subject effects.

**Supplementary Information:**

The online version contains supplementary material available at 10.1007/s10899-023-10191-5.

## Introduction

According to the fifth edition of the Diagnostic and Statistical Manual of Mental Disorders (DSM-5), gambling disorder (GD) is an addictive disorder characterized by problematic gambling behavior that causes significant impairment manifested in symptoms of (i) tolerance, (ii) withdrawal symptoms, (iii) unsuccessful attempts to control gambling, (iv) preoccupation with gambling, (v) gambling if having negative affective states, (vi) chasing, (vii) lying related to gambling behavior, (viii) risking or losing relationships, work- or educational-related attainments, and (ix) financially relying on others due to gambling (American Psychiatric Association, 2013). However, gambling problems or harms can be more broadly conceptualized from an epidemiological perspective. That is, gambling can lead to financial and relationship problems, psychological or physical health problems, problematic work or school performance, diminished contribution to cultural life or community, and criminal activities. Moreover, this approach stresses that not only individuals with GD experience gambling-related problems or harms but also low- or at-risk gamblers experience the potentially detrimental effects of gambling at the levels of family, social network, and society (Langham et al., 2015). A previous meta-analytic study reported that prevalence rates of past-year problem gambling among adults vary between 0.1% and 5.8% (Calado & Griffiths, 2016). However, young adults can show an elevated risk for problem gambling (Nowak, 2018); therefore, further examination of gambling problems might be warranted in this risk group.

The network approach has been used increasingly in the past few years to examine symptom structures of psychopathologies. This framework conceptualizes mental disorders as a system of causally interrelated symptoms. The network approach can identify symptoms strongly linked to other symptoms that have high importance and centrality in a network (Granero et al., 2021). Therefore, the network approach can generate hypotheses regarding symptoms that play important roles in the development or progression of mental disorders (Contreras et al., 2019; Fried et al., 2017). The assumption of causally related symptoms can complement theoretical models of addictive disorders, which describe the step-by-step and cyclical progression and maintenance of addictive behaviors (for review, see Demetrovics et al., 2022; Perales et al., 2020). For example, a problem gambler might first experience the symptom of impaired control over use which might subsequently lead to heightened preoccupation and tolerance to the gambling behavior, which can further contribute to the development of withdrawal symptoms in addition to problems in social functioning and financial status (Demetrovics et al., 2022; Perales et al., 2020; Rhemtulla et al., 2016).

Multiple studies have used the network approach to examine addictive behaviors, for example, by exploring symptom networks of addictive behaviors (Conlin et al., 2022; Huth et al., 2022; Rhemtulla et al., 2016; Svicher et al., 2021), and co-occurrence patterns or risk factors of addictive disorders (Anker et al., 2017; Baggio et al., 2018b, 2018c; Huang et al., 2021; Xia et al., 2021). Regarding problem gambling, some studies have also attempted to explore networks of risk characteristics among individuals with GD by considering sociodemographic factors, age of onset and duration of GD, personality traits, addictive behaviors, and psychological distress (Granero et al., 2021). However, only a few of the existing network analytical papers investigated the centrality of symptoms of problem gambling (Baggio et al., 2016; Baggio et al., 2018a; Rozgonjuk et al., 2021; Zarate et al., 2022). These studies have explored the co-occurrence and symptomatic links between addictive behaviors (including gambling) (Baggio et al., 2016; Rozgonjuk et al., 2021; Zarate et al., 2022). Moreover, Baggio et al. (2018a) explored a network of gambling activities and symptoms of problem gambling. Although these studies differed in symptom centrality because they incorporated diverse constructs and measurements, they converged partially on symptoms that showed high centrality in the estimated networks. The symptoms of prioritizing gambling over other duties or areas, feeling guilty when gambling, feeling as having a problem with gambling, being criticized by others, having impaired control over gambling, and asking for money due gambling showed high centrality (Baggio et al., 2018a; Rozgonjuk et al., 2021; Zarate et al., 2022).

It is important to mention that not just network analysis can be used to assess the importance of problem gambling symptoms. Previous studies used also principal component analysis (PCA) or item response theory (IRT) to show that the information capacity of problem gambling symptoms varies as a function of problem gambling severity. For example, in an adult community sample, it was demonstrated that chasing losses, lying, escapism, and preoccupation are frequently endorsed symptoms among gamblers who do not reach the threshold of GD, while more severe GD cases are characterized by the heightened presence of loss of control, withdrawal symptoms, tolerance, negative social and financial consequences, and illegal acts (the last symptom was observed among gamblers with the most severe GD) (Toce-Gerstein et al., 2003). Moreover, other IRT-based results (in adult community samples) suggested that symptoms of salience, borrowing money/financial problems, deception, health problems, and being criticized by others because of gambling have high discriminative capacity at more severe levels of gambling problems (Gorenko & Konnert, 2022; Stavropoulos et al., 2022).

However, the previous findings on symptomatic networks of problem gambling were all based on cross-sectional data. The main limitation of cross-sectional symptom networks is that they cannot be used to test directional relationships and temporal order between symptoms (Forbes et al., 2017a, 2017b). Therefore, exploring symptomatic longitudinal networks can increase the current knowledge of networks of problem gambling symptoms. Moreover, previous studies have repeatedly raised that cross-sectional symptom networks have limited replicability (e.g., the difference across studies in which symptom is the most central) ﻿(Forbes et al., 2017a, 2017b). None of the previous studies which included problem gambling symptoms have attempted to test the replicability of symptom networks between different independent samples, different classes of gamblers, or different time points. Therefore, it is necessary to assess the reproducibility of cross-sectional networks of problem gambling symptoms to determine their generalizability.

### Aims

The aims of the present study were twofold. The first aim was to examine cross-sectional networks of problem gambling symptoms and evaluate their replicability (i.e., symptoms with the highest centrality levels, correlations between the cross-sectional networks in the presence vs. absence of edges, rank order of edge weights, and centrality estimates, and network invariance tests between the cross-sectional networks). The convergence of cross-sectional networks was investigated among young adult gamblers from a representative sample across two-time points. The second aim was to explore a longitudinal network of problem gambling symptoms using the cross-lagged panel network (CLPN) framework (Rhemtulla et al., 2022). Previous studies have applied the CLPN framework to examine longitudinal symptom networks of an alcohol use disorder, major depression, or post-traumatic stress disorder (Conlin et al., 2022; Funkhouser et al., 2021; Rubin et al., 2021). It is important to note that this approach does not control simultaneously for between- and within-person differences; therefore, causality between the symptoms cannot be evaluated (Conlin et al., 2022; Epskamp, 2020; Hamaker et al., 2015). However, such a directed network could increase the current knowledge and provide new insights into between-person or group-level differences in symptom networks of problem gambling.

## Method

### Participants and Procedures

Participants in the present study were drawn from the first two waves of the Budapest Longitudinal Study (BLS). BLS aimed to examine longitudinal patterns and psychosocial correlates of addictive behaviors. Young adults born between 1984 and 2000 in Budapest (the capital city of Hungary) were the study's target population. Random and stratified sampling was performed by age groups and districts of Budapest. The initial sample was selected based on the Hungarian Interior Ministry’s register of young adults born between 1984 and 2000 with a residential address in Budapest. The interviewers first tried to reach the participants via an invitation letter and later attempted to contact the respondents. If the participants could not be contacted successfully on three occasions, refused to respond, or could not participate (e.g., long-term absence), matched samples were recruited to compensate for the loss of participants. Wave 1 data were collected between March and July 2019 and Wave 2 data between June and September 2020. A mixed, in-person survey method was applied in the study, combining face-to-face surveying (e.g., socio-demographics, screening questions for addictive behaviors) and self-report questionnaires (e.g., psychoactive substance use, behavioral addictions, psychological characteristics). Moreover, online interview and self-report methods were also utilized in Wave 2. All individuals provided informed consent for their voluntary participation in the study. The Research and Ethical Committee of the Medical Research Council approved the study protocol of the BLS (no. 60471–2/2018/EKU).

The net sample size was 3890 in Wave 1 (age: *M* = 27.06 [*SD* = 4.76]; female gender: 51.58% [*N* = 2007]) and 2801 in Wave 2 (age: *M* = 28.03 [*SD* = 4.77]; female gender: 51.97% [*N* = 1456]). Overall, 2777 individuals participated in both waves, 1113 individuals dropped out after Wave 1, and 24 individuals participated only in Wave 2. Attrition after Wave 1 was due to the unavailability of participants, refusal of participation, and not filling out the online questionnaire. Moreover, participants were excluded from the net samples after quality control of responses. For the final analyses, only data from individuals who (i) reported past-year gambling at both study waves and (ii) had valid responses on all items of the Problem Gambling Severity Index (PGSI) at both study waves were considered. Thus, the final sample included 335 young adult gamblers (age in Wave 2: *M* = 29.67 [*SD* = 4.12]; female gender: 45.97% [*N* = 154]). In other words, (i) 699 individuals were excluded as they reported about past-year gambling only in Wave 1 or Wave 2 but not both waves, (ii) 1171 individuals were excluded because they did not report about past-year gambling at either study wave (e.g., lifetime non-gamblers reported about gambling in their lifetime but not during the past year), (iii) 491 individuals were excluded due to inconsistent responses (i.e., lifetime or past-year gambling in Wave 1 but reported lifetime absence of gambling in Wave 2), and (iv) 81 participants were excluded due to missing responses on the gambling screening questions and items of the PGSI.

### Measures

Symptoms of problem gambling were measured with the 9-item Problem Gambling Severity Index (PGSI) (Ferris & Wynne, 2001; Gyollai et al., 2013). Each item on the scale was rated on a four-point scale (0 = Never, 3 = Almost always). Based on the total PGSI score, four risk categories were defined: (i) non-problematic gamblers (0 pts), (ii) low-risk gamblers (1–2 pts), (iii) moderate-risk gamblers (3–7 pts), and (iv) severe problem gamblers (8 or more pts). However, due to the very high proportions of ‘Never’ responses in Waves 1–2 (92.84–97.31%; Table 1), items of the PGSI were dichotomized to conduct network analysis (0 = Never, 1 = At least sometimes) and avoid zero cell counts across response categories during the estimation of polychoric correlations. Internal consistencies of the PGSI were *α* = 0.94 in Wave 1 and *α* = 0.98 in Wave 2.

**Table 1:** Prevalence rates of problem gambling symptoms and gambling risk categories

	Wave 1			Wave 2		
Problem gambling symptom *%* (*N*)	Total sample (*N* = 335)	Females (*N* = 154)	Males (*N* = 181)	Total sample (*N* = 335)	Females (*N* = 154)	Males (*N* = 181)
1. Betting more than one can afford	4.78% (*N* = 16)	1.30% (*N* = 2)	7.73% (*N* = 14)	7.16% (*N* = 24)	7.14% (*N* = 11)	7.18% (*N* = 13)
2. Tolerance	5.67% (*N* = 19)	2.60% (*N* = 4)	8.29% (*N* = 15)	5.97% (*N* = 20)	5.84% (*N* = 9)	6.08% (*N* = 11)
3. Chasing losses	6.87% (*N* = 23)	3.90% (*N* = 6)	9.39% (*N* = 17)	5.37% (*N* = 18)	5.19% (*N* = 8)	5.52% (*N* = 10)
4. Borrowing money	2.69% (*N* = 9)	1.30% (*N* = 2)	3.87% (*N* = 7)	5.37% (*N* = 18)	4.55% (*N* = 7)	6.08% (*N* = 11)
5. Recognizes one has a problem	3.88% (*N* = 13)	2.60% (*N* = 4)	4.97% (*N* = 9)	5.08% (*N* = 17)	5.84% (*N* = 9)	4.42% (*N* = 8)
6. Health problems	3.28% (*N* = 11)	3.25% (*N* = 5)	3.31% (*N* = 6)	5.37% (*N* = 18)	5.84% (*N* = 9)	4.97% (*N* = 9)
7. Criticized by others	3.58% (*N* = 12)	2.60% (*N* = 4)	4.42% (*N* = 8)	5.08% (*N* = 17)	5.19% (*N* = 8)	4.97% (*N* = 9)
8. Financial problems	2.69% (*N* = 9)	1.30% (*N* = 2)	3.87% (*N* = 7)	6.27% (*N* = 21)	5.84% (*N* = 9)	6.63% (*N* = 12)
9. Feelings of guilt	4.78% (*N* = 16)	2.60% (*N* = 4)	6.63% (*N* = 12)	6.27% (*N* = 21)	5.84% (*N* = 9)	6.63% (*N* = 12)
Gambling risk categories *%* (*N*)						
Non-problematic gamblers	89.25% (*N* = 299)	92.86% (*N* = 143)	86.19% (*N* = 156)	90.45% (*N* = 303)	92.21% (*N* = 142)	88.95% (*N* = 161)
Low-risk gamblers	6.27% (*N* = 21)	4.55% (*N* = 7)	7.73% (*N* = 14)	2.99% (*N* = 10)	1.95% (*N* = 3)	3.87% (*N* = 7)
Moderate risk gamblers	2.09% (*N* = 7)	1.30% (*N* = 2)	2.76% (*N* = 5)	2.09% (*N* = 7)	0.65% (*N* = 1)	3.31% (*N* = 6)
Severe problem gamblers	2.39% (*N* = 8)	1.30% (*N* = 2)	3.31% (*N* = 6)	4.48% (*N* = 15)	5.19% (*N* = 8)	3.87% (*N* = 7)
Problem Gambling Severity Index (PGSI) total score *M* (*SD)*	0.50 (2.21)	0.27 (1.60)	0.69 (2.61)	0.82 (3.25)	0.88 (3.56)	0.77 (2.97)

Numbers related to problem gambling symptoms are used as abbreviations in figures of network analyses

### Data Analysis

A series of network analyses were performed to explore cross-sectional and cross-lagged, longitudinal symptom networks of problem gambling. As an initial step, separate cross-sectional networks of problem gambling symptoms were estimated in Wave 1 and Wave 2. The undirected networks contained nine nodes of problem gambling symptoms, and 36 edge weights were estimated between them. As dichotomous variables measured these symptoms, the Ising model estimation method was used (van Borkulo et al., 2015). This technique builds on node-based binary logistic regression with the least absolute shrinkage and selection operator (LASSO) regularization procedure and uses the extended Bayesian information criterion (EBIC) for model selection. To obtain parsimonious and sparse network models, the EBIC hyperparameter’s value was specified at 0.25, and the ‘AND-rule’ was applied (i.e., an edge between two given nodes [n_1_ and n_2_] can only be non-zero if both regression effects of n_1_ on n_2_ and n_2_ on n_1_ are non-zero) (van Borkulo et al., 2015). The standardized centrality measures of strength, closeness, and betweenness were calculated to quantify the importance of problem gambling symptoms in the networks. The strength indicator measures a node’s direct effect on the other nodes in the network (i.e., the sum of edge weights in absolute values related to each node in the network). Higher values on the closeness index can indicate a node’s tight association with other nodes and show that changes in other nodes have increased direct or indirect effects on that node (i.e., the sum of the inverse of distances related to each node in the network). The index of betweenness quantifies a node’s transmitting role between other nodes (i.e., summed frequency of being located in the shortest path between two other nodes) (Costantini et al., 2015; Epskamp & Fried, 2018; Epskamp et al., 2018).

Next, we examined the congruence and replicability of cross-sectional networks between Wave 1 and Wave 2. Phi correlations (*φ*) indicated the associations between the presence vs. absence of edges. Spearman’s rank correlation (*ρ*) was calculated between edge weights and node-based centrality measures. Moreover, the invariance of the cross-sectional networks was assessed by testing the maximum difference in any of the edge weights between the two networks and by testing the difference in global strength between the two networks. Previous studies have used a similar approach to assess the replicability of networks (Conlin et al., 2022).

A longitudinal network of problem gambling symptoms was explored utilizing the CLPN framework (Rhemtulla et al., 2022). First, nine binary logistic regression models with LASSO regularization were specified to examine autoregressive and cross-lagged symptomatic effects. The predictors were the dichotomous problem gambling symptoms in Wave 1 (same in all models), and the outcome variables were the dichotomous gambling symptoms in Wave 2 (different outcomes in each model). Each model was estimated using tenfold cross-validation based on the deviance statistic to determine the optimal lambda (*λ*) penalty parameter. The values of *λ* were largest within one standard error of the minimum *λ*. This technique can be employed to obtain the simplest models with the fewest non-zero parameters. Next, the autoregressive and cross-lagged regression estimates were used as input for the longitudinal cross-lagged network analysis with nine nodes and 81 edge weights. Previous studies have used a similar approach to estimate cross-lagged symptomatic networks (Rubin et al., 2021). The standardized out-strength and in-strength centrality estimates were calculated to assess the importance of problem gambling symptoms in the longitudinal network. Out-strength sums the direct effect of a node in Wave 1 on other nodes in Wave 2, whereas in-strength quantifies the direct effects on a node in Wave 2 by other nodes in Wave 1.

The stability and accuracy of the cross-sectional and cross-lagged network parameters were investigated as supplementary analyses. These validation analyses were performed with 1000 random bootstrap samples. First, non-parametric bootstrapped confidence intervals (CIs) were calculated for edge weights to assess their accuracy. Second, non-parametric bootstrapped difference tests were computed to assess differences between nodes in centrality estimates and edge weights. Third, the stability of centrality indices was measured with a case-dropping subset bootstrap analysis to obtain high correlation stability (CS) coefficients which can reveal a high proportion (preferably at least 50%) of the cases that can be dropped (with a 95% probability) to retain at least 0.70 correlations for centrality measures between the full sample and case-dropping subsets (Epskamp et al., 2018).

Cross-sectional and longitudinal network estimation and calculation of centrality measures were performed using the Bootnet (Epskamp et al., 2018) and Qgraph (Epskamp et al., 2012) R packages. The Psych (Revelle, 2011) and NetworkComparisonTest (van Borkulo et al., 2022) R packages were used for the cross-sectional network comparisons. Cross-lagged binary logistic LASSO regression models were estimated using the Glmnet R package (Hastie et al., 2022). The stability and accuracy of the network parameters were examined with the Bootnet R package. Dataset and codes for the analyses are available at https://osf.io/adn3m/.

## Results

### Descriptive Statistics

Table 1 presents the prevalence rates of each problem gambling symptom and gambling risk categories in Waves 1–2 in the total sample and for females and males separately. Generally, prevalence rates of each gambling symptom were very low, and approximately nine out of ten gamblers did not experience any gambling problems. The PGSI total score did not change significantly across waves in the total sample (mean dependent difference: *t* [334] = 1.74; *p* = 0.084; *d*_Pooled_ = 0.08; dependent correlation: *r* = 0.26; *p* < 0.001) and among males (mean dependent difference: *t* [180] = 0.35; *p* = 0.724; *d*_Pooled_ = 0.02; dependent correlation: *r* = 0.37; *p* < 0.001). However, a significant but small increase in the PGSI total score was shown for females from Wave 1 to Wave 2 (mean dependent difference: *t* [153] = 2.03; *p* = 0.044; *d*_Pooled_ = 0.12; dependent correlation: *r* = 0.12; *p* = 0.145). Non-significant dependent associations were found in the total sample (standard McNemar test: *χ*^*2*^ [1] = 0.36; *p*_Standard_ = 0.546; *p*_Binomial_ = 0.651; *r* = 0.07) as well as among males (standard McNemar test: *χ*^*2*^ [1] = 0.81; *p*_Standard_ = 0.369; *p*_Binomial_ = 0.473; *r* = 0.07) and females (standard McNemar test: *χ*^*2*^ [1] = 0.08; *p*_Standard_ = 0.782; *p*_Binomial_ > 0.999; *r* = 0.02) when comparing the paired proportions of ‘non-problematic gamblers’ vs. ‘at least low-risk gamblers.’

### Cross-Sectional Networks

**Fig. 1:**
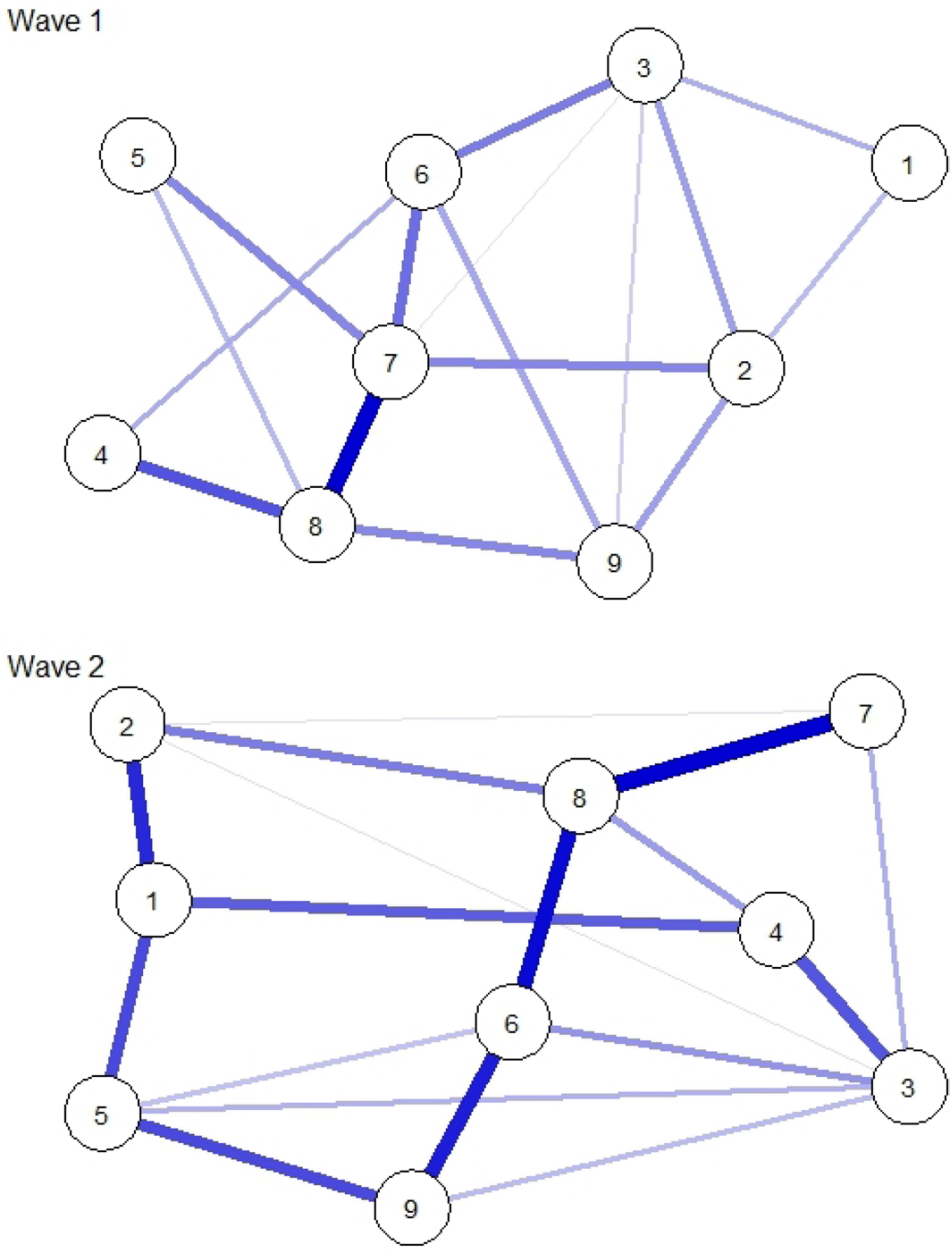
Cross-sectional networks of problem gambling symptoms in Waves 1 and 2. The blue colored lines indicate positive associations between the nodes, and thicker lines indicate stronger relationships between the nodes. Node abbreviations: 1—Betting more than one can afford, 2—Tolerance, 3—Chasing losses, 4—Borrowing money, 5—Recognizes one has a problem, 6—Health problems, 7—Criticized by others, 8—Financial problems, 9—Feelings of guilt

Cross-sectional networks of problem gambling symptoms in Waves 1 and 2 are displayed in Fig. 1. Supplementary Table 1 presents the edge weights of the cross-sectional networks. Sixteen (44.44%) edges were positive and non-zero with a mean non-zero edge weight of *b* = 2.67 in Wave 1, and 17 edges (47.22%) were positive and non-zero with a mean non-zero edge weight of *b* = 2.90 in Wave 2. The strongest positive association was shown between symptoms of ‘Criticized by others’ (7) and ‘Financial problems’ (8) in both waves.

**Fig. 2:**
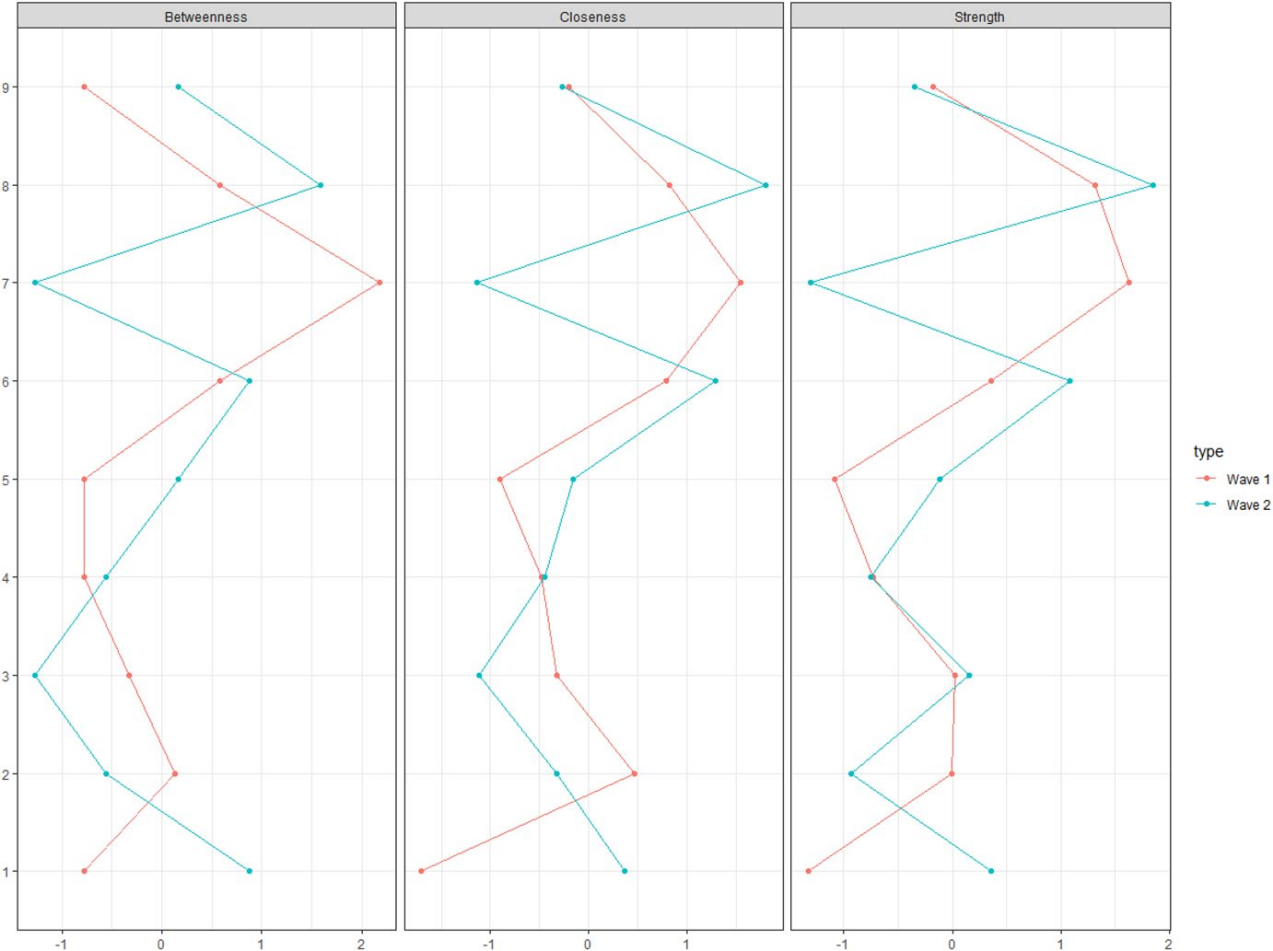
Standardized centrality indices based on cross-sectional networks of problem gambling symptoms from Waves 1 and 2. Node abbreviations: 1—Betting more than one can afford, 2—Tolerance, 3—Chasing losses, 4 – Borrowing money, 5 – Recognizes one has a problem, 6—Health problems, 7—Criticized by others, 8—Financial problems, 9—Feelings of guilt

Standardized centrality estimates of the cross-sectional networks are shown in Fig. 2. A divergent pattern of nodes with the highest centrality estimates was found between Waves 1 and 2. ‘Criticized by others’ (7) was the most central symptom across all indices in Wave 1; however, it was the least central symptom on all indices in Wave 2. Overall, ‘Financial problems’ (8) was the most central symptom across all indices in Wave 2.

Low levels of congruity were shown between cross-sectional networks in Wave 1 and Wave 2 in terms of edges and centrality indices. Non-significant, positive, and small rank correlations were shown in the presence of edges (*φ* = 0.16; *p* = 0.346) and edge weights (*ρ* = 0.12; *p* = 0.475). Marginal and close-to-zero correlations were shown in the node-based rank order of the centrality indices (betweenness: *ρ* = -0.08, *p* = 0.847; closeness: *ρ* = -0.05, *p* = 0.912; strength: *ρ* = 0.00, *p* > 0.999). However, network invariance tests indicated non-significant maximum differences in any of the edge weights (M = 5.21; *p* = 0.82) and global strength (S = 6.53; *p* = 0.68) between the two cross-sectional networks.

Supplementary Figures 1–4 show the results of the stability and accuracy analyses of the cross-sectional network parameters. Wide bootstrapped CIs indicated the low accuracy of most edge weights (Supplementary Figure 1). Non-significant differences were shown between nodes on all centrality estimates (Supplementary Figure 2) and between edge weights (Supplementary Figure 3) in both waves. Decreased stability of the centrality estimates was indicated by low CS-coefficients (0.00–0.05) and by considerable decreases in correlations of centrality indices between the original sample and subsets (Supplementary Figure 4). These findings suggest that the rank order of edge weights and nodes in terms of centrality measures should be interpreted carefully.

### Cross-Lagged Longitudinal Network

**Fig. 3:**
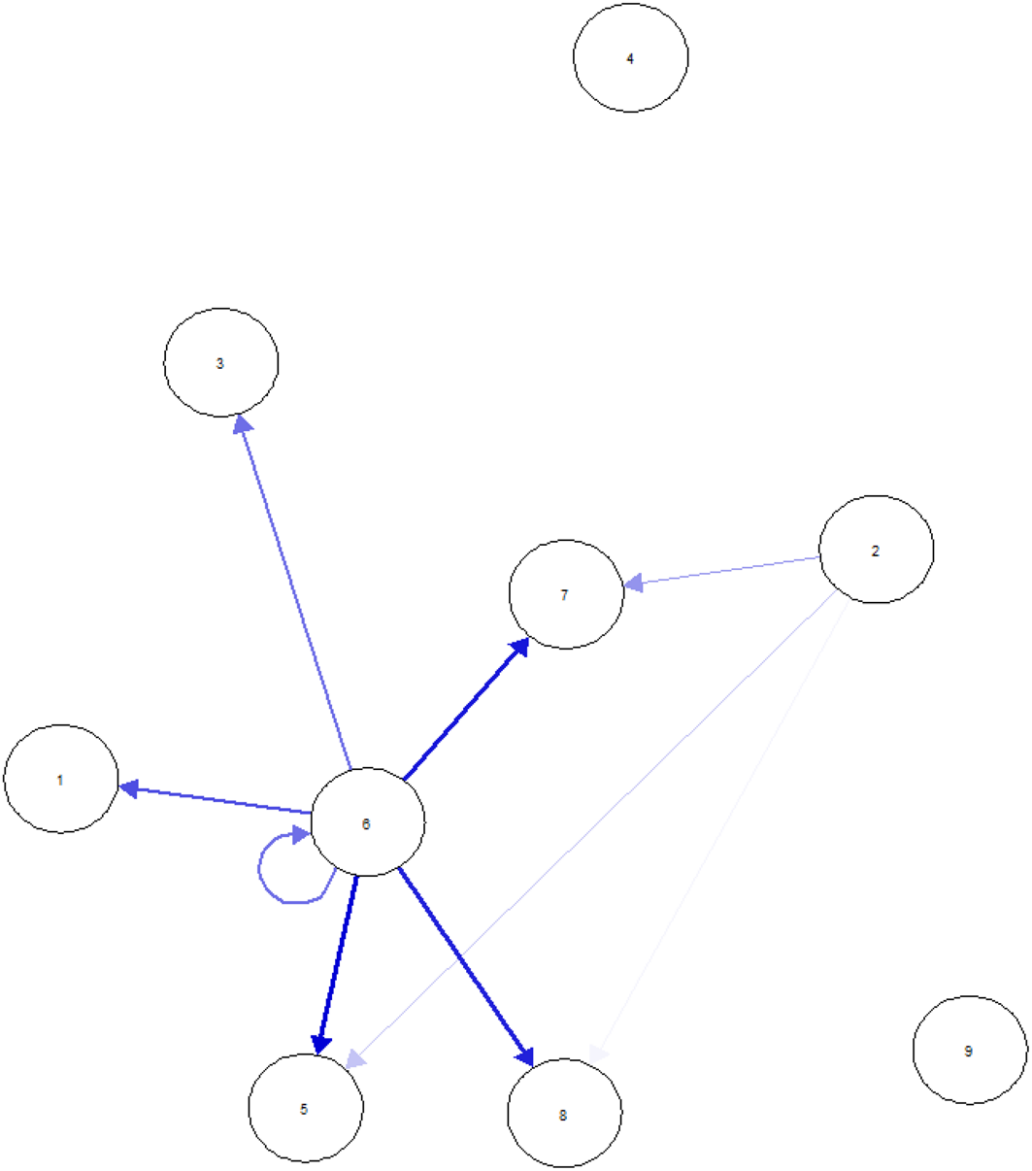
Longitudinal cross-lagged network of problem gambling symptoms between Waves 1 and 2. The blue colored lines indicate positive associations between the nodes, and thicker lines indicate stronger relationships between the nodes. The arrows show the predictive effects of symptoms in Wave 1 on symptoms in Wave 2. Loop arrows represent autoregressive effects. Node abbreviations: 1—Betting more than one can afford, 2—Tolerance, 3—Chasing losses, 4—Borrowing money, 5—Recognizes one has a problem, 6—Health problems, 7—Criticized by others, 8 – Financial problems, 9—Feelings of guilt

The cross-lagged longitudinal network of problem gambling symptoms between Waves 1 and 2 is displayed in Fig. 3. Cross-lagged and autoregressive edge weights are summarized in Supplementary Table 2. Nine (11.11%) edge weights were positive and non-zero, with the mean non-zero edge weight of *b* = 0.98. The presence of ‘Tolerance’ (2) in Wave 1 positively predicted the presence of ‘Recognizes one has a problem’ (5), ‘Criticized by others’ (7), and ‘Financial problems’ (8) symptoms in Wave 2. The presence of ‘Health problems’ (6) in Wave 1 positively predicted the presence of ‘Betting more than one can afford’ (1), ‘Chasing losses’ (3), ‘Recognizes one has a problem’ (5), ‘Health problems’ (6), ‘Criticized by others’ (7), and ‘Financial problems’ (8) symptoms in Wave 2.

**Fig. 4:**
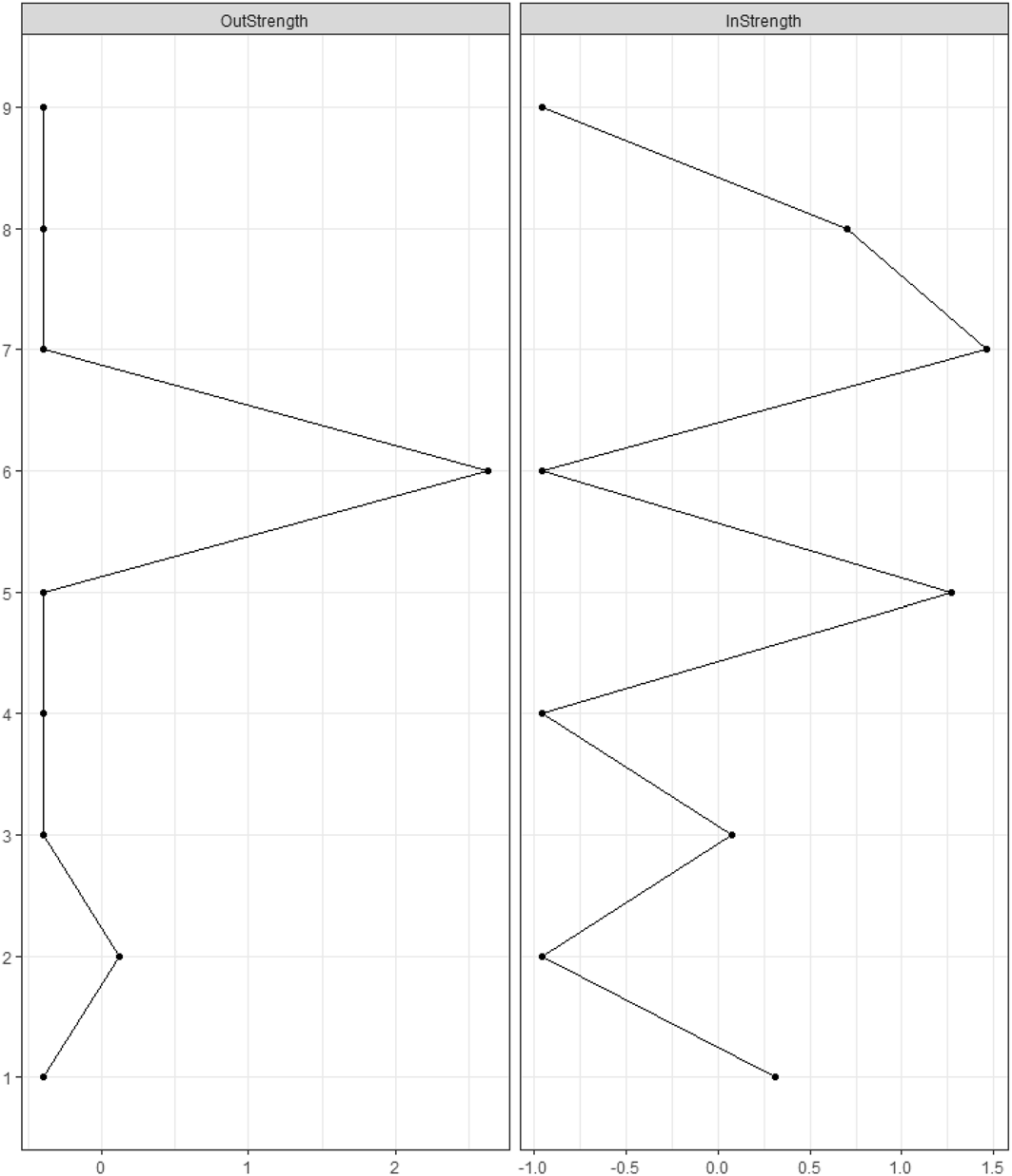
Standardized centrality indices based on the longitudinal cross-lagged network of problem gambling symptoms between Waves 1 and 2. Node abbreviations: 1—Betting more than one can afford, 2—Tolerance, 3—Chasing losses, 4 – Borrowing money, 5—Recognizes one has a problem, 6—Health problems, 7—Criticized by others, 8—Financial problems, 9—Feelings of guilt

Standardized centrality estimates based on the cross-lagged longitudinal network are shown in Fig. 4. The highest out-strength was shown for ‘Health problems’ (6), followed by ‘Tolerance’ (2). The highest in-strength was shown for ‘Criticized by others’ (7), followed by ‘Recognizes one has a problem’ (5) and ‘Financial problems’ (8).

Supplementary Figs. 5–7 present the accuracy of the cross-lagged network parameters. Non-zero edges from the symptom of ‘Health problems’ (6) were non-reproducible in the bootstrap samples. Non-zero edges from the symptom of ‘Tolerance’ (2) on ‘Recognizes one has a problem’ (5) and ‘Criticized by others’ (7) were non-zero and positive also for the bootstrap samples in addition to other non-zero edges in the bootstrap samples (which were originally zero) from ‘Tolerance’ (2) (i.e., on symptoms 1, 3, 6, and 9) (Supplementary Fig. 5). The non-zero predictive effects of symptoms of ‘Health problems’ (6) and ‘Tolerance’ (2) estimated in the sample were significantly stronger compared to multiple edge weights; however, significant differences were shown for the non-zero edges from ‘Tolerance’ (2) (Supplementary Fig. 6). The symptom of ‘Health problems’ (6) presented significantly higher out-strength compared to symptoms of ‘Betting more than one can afford’ (1), ‘Tolerance’ (2), and ‘Chasing losses’ (3), whereas ‘Tolerance’ had significantly higher out-strength compared to all other nodes (except for ‘Health problems’ [6]). The highest in-strength was shown for ‘Criticized by others’ (7) compared to all other nodes (Supplementary Table 7). Very low stability was indicated for in-strength, while an adequate level was demonstrated for out-strength (i.e., CS-coefficients: 0.00 and 0.28, respectively).

## Discussion

Overall, evidence for the replicability of cross-sectional symptom networks of problem gambling is limited. A divergent pattern was shown between the study waves in symptoms with the highest centrality levels. On the one hand, being criticized by others was the most central symptom in Wave 1 but had the lowest centrality in Wave 2. On the other hand, financial problems due to gambling were the most central symptom in Wave 2 and the second most central symptom in Wave 1. Previous studies have also highlighted the potentially central roles of financial problems/asking for money in cross-sectional symptom networks of problem gambling (Zarate et al., 2022). Moreover, other findings (not based on network analysis) have shown that the presence of financial problems/borrowing money for gambling could be indicative of higher severity of problem gambling and associated with treatment-seeking behavior (Gorenko & Konnert, 2022; Stavropoulos et al., 2022; Toce-Gerstein et al., 2003; Valdivia-Salas et al., 2014). However, the findings of the sensitivity analyses indicated very low stability and accuracy of the parameters of the cross-sectional networks; therefore, the above patterns and differences should be interpreted cautiously.

Moreover, the cross-sectional symptom networks showed only fractional congruence with each other in terms of edges and centrality indices. On the one hand, low congruence was shown for the presence vs. absence of edges and rank order of the edge weights between the two study waves. Moreover, correlations between cross-sectional networks in terms of the node-based rank order of centrality estimates were lacking. These findings are consistent with previous findings indicating low replicability of cross-sectional symptom networks (Forbes et al., 2017a, 2017b). The present study suggests that cross-sectional symptom networks of problem gambling can change over time, leading to different inferences on the importance of symptoms. Therefore, clinical implications from cross-sectional networks of problem gambling symptoms (e.g., the most central symptom can serve as a possible intervention target) should be drawn with great care (Forbes et al., 2017a, 2017b). However, the small sample size of the present study, and in particular the very small number of participants with severe problem gambling in both study waves, may have contributed to the limited reproducibility of the cross-sectional symptom networks.

Moreover, it is important to note that Wave 2 data collection was carried out around the first and second waves of the COVID-19 pandemic. Although the present study did not control for COVID-19 pandemic-related factors, it might be possible that COVID-19 might have partly affected the observed changes between the cross-sectional networks. Several studies have examined the changes in gambling behavior due to the COVID-19 pandemic (Brodeur et al., 2021; Hodgins & Stevens, 2021), reporting a decrease in overall gambling frequency and expenditure. This pattern may be associated with the closure of physical venues during the lockdown, the limited availability of sports betting due to canceled sporting events, financial reasons (e.g., the person had less available money for gambling), and gambling-related characteristics (e.g., decreased interest/desire to gamble, not wanting to gamble around family, perception of high levels of gambling) (Brodeur et al., 2021; Hodgins & Stevens, 2021). However, other findings indicated changes in preferred gambling type (e.g., increases in online casino or horse betting), which may also influence the networks of problem gambling (Brodeur et al., 2021). In addition, some studies have identified a risk subgroup of individuals who showed increased gambling behavior during the COVID-19 pandemic. These individuals tended to be younger and male, have higher levels of problem gambling and poorer mental health status (e.g., depression, anxiety symptoms), and report higher levels of substance use. Individuals who gambled more during the COVID-19 pandemic frequently cited financial (e.g., winning money, financial pressure) and boredom-related reasons (Brodeur et al., 2021; Hodgins & Stevens, 2021). Therefore, changes in gambling motivations during the COVID-19 pandemic may have influenced the results of the present study. It is important to note that a few studies have examined the potential effect of the COVID-19 pandemic on problem gambling symptoms. For example, low prevalence rates of various problem gambling symptoms and reduced gambling cravings were observed among individuals treated for problem gambling (except for having a gambling problem and feeling guilty because of gambling) (Donati et al., 2021).

On the other hand, other statistical indices suggested higher congruence between the cross-sectional networks. Specifically, the network invariance tests between the two cross-sectional networks indicated non-significant maximum differences between edges and non-significant differences in global network strength. Overall, these findings might imply a high similarity between cross-sectional networks at the global network level (e.g., number of non-zero edges, summed edge weights, maximum differences between edges) than at the node-specific level.

The present study also attempted to explore cross-lagged symptomatic networks of problem gambling for the first time. The longitudinal model demonstrated that the presence of health problems due to gambling (e.g., stress, anxiety) and tolerance in the first wave positively predicted multiple problem gambling symptoms in the second wave of the study. Previous studies and theoretical models have also highlighted the role of tolerance. For example, in previous studies, tolerance (along with other problem gambling symptoms) was identified as a criterion that can differentiate between social and problem gamblers (Temcheff et al., 2016) and characterize gamblers with a clinical level of GD (Toce-Gerstein et al., 2003). Process-based addiction models suggest that neuroadaptation processes can explain tolerance, which can play a central role in the shift from positively reinforcing (e.g., gambling due to the excitement) to negatively reinforcing motives for gambling (e.g., gambling to cope with withdrawal symptoms) (Blaszczynski et al., 2008; Perales et al., 2020). However, the literature has questioned the validity of the criterion for tolerance applied in the present study (i.e., needed to gamble with larger amounts of money to get the same feeling of excitement). Namely, instead of the neuroadaptation-based addiction process model, a multidimensional concept of gambling tolerance was suggested, which includes the excitement related to an uncertain outcome of winning vs. losing money, the excitement related to perceived risk vs. safety, and maladaptive beliefs on probability of winning (Blaszczynski et al., 2008; Lee et al., 2020). Therefore, the present study might have measured the symptom of tolerance inappropriately.

It is important to note that health problems due to gambling are not included in the diagnostic criteria of GD (American Psychiatric Association, 2013). However, taxonomies of harmful gambling cover gambling’s detrimental effects on psychological and physical health, including various negative health consequences (e.g., increased distress, shame, stigmatization, decreased self-esteem, increased blood pressure, and reduced sleeping). Harmful consequences on health can occur directly as a result of gambling, but it is also possible that the health effects are associated and interact with other harmful effects of gambling (Langham et al., 2015). Thus, it might be possible that the predictive effects of health problems on other problem gambling symptoms indicate bidirectional processes between problem gambling symptoms. Moreover, a previous study using IRT showed that the presence of health problems due to gambling has a high discriminative capacity at more severe levels of problem gambling (Gorenko & Konnert, 2022). However, it is very important to consider that the cross-lagged network of problem gambling symptoms did not account for the within-subject changes; thus, any causal inferences should be avoided. Furthermore, the low overall sample size combined with the small number of severe problem gamblers in the study may have biased symptoms’ predictive effects; therefore, the cross-lagged analysis should be interpreted with caution.

### Limitations

The present findings should be interpreted with care due to various methodological limitations. First, it was impossible to explore causal and bidirectional associations between problem gambling symptoms and within-subject changes in the cross-sectional and cross-lagged networks. Second, the PGSI measured symptoms of problem gambling only superficially and non-comprehensively; for example, some symptoms of GD (e.g., lying related to gambling behavior) and gambling-related harms (e.g., relationship conflicts) were not included in the network models. In addition to these, other gambling elements could have been included as well (e.g., preference for gambling types). Third, the self-reported measurement of problem gambling symptoms might have biased the present findings (e.g., misunderstanding questionnaire items, concealing gambling problems). Fourth, a relatively high proportion of the (excluded) participants responded to the gambling screening questions inconsistently; for example, by reporting lifetime or past-year gambling in Wave 1 but reporting lifetime absence of gambling in Wave 2. This might have underestimated the prevalence of problem gambling symptoms and limited the replicability of cross-sectional symptom networks. Fifth, exploring the symptom networks more precisely was difficult because of the low prevalence rates and the binary measurement of problem gambling symptoms. In line with this, this study's conclusions should be interpreted with caution due to the potential bias arising from the low overall sample size and the small number of participants with severe problem gambling. Finally, the sensitivity analyses indicated low stability and accuracy of the estimated network parameters; therefore, the differences between problem gambling symptoms should also be interpreted cautiously.

## Conclusions

The present findings provide limited evidence for the replicability of cross-sectional symptom networks of problem gambling. The congruence between the cross-sectional networks in the symptoms’ importance, the presence of non-zero edges, and the rank order of edge weights and different centrality measures was minimal. Thus, further research might need to clarify the role of centrality measures in interpreting symptom networks and the use of cross-sectional network analyses in generating hypotheses in the context of problem gambling. The present study also explored a longitudinal CLPN of problem gambling symptoms and suggested that the symptoms of tolerance and health problems might predict subsequent problem gambling symptoms. This approach might provide a more accurate view of the group-level differences in a symptom network of problem gambling, though not controlling for within-subject changes limits the generalizability of the findings. To assess causal relationships between problem gambling symptoms more accurately, future research should use a more extensive longitudinal design (i.e., more measurement points) with larger samples, including more problem gamblers, and analytical methods that can specifically distinguish within- and between-subject effects. Such network models could identify problem gambling symptoms that play a crucial role in the progression and maintenance of problem gambling and can be targets of intervention.

### Supplementary Information

Below is the link to the electronic supplementary material.

Supplementary file1 (DOCX 839 kb)
