# Supplementary material for: The Predictive Role of Tolerance and Health Problems in Problem Gambling: A Cross-Sectional and Cross-Lagged Network Analyses
Source: J Gambl Stud. 2023 Feb 4;39(4):1781–98. doi: 10.1007/s10899-023-10191-5 (PMC9898861; doi:10.1007/s10899-023-10191-5)
Supplement: Supplementary file 1 — Supplementary file1 (DOCX 839 kb) [file 10899_2023_10191_MOESM1_ESM.docx]

**Supplementary materials**

Supplementary Table 1. Edge weights of the cross-sectional networks of problem gambling symptoms from Waves 1 and 2.

|  | **1** | **2** | **3** | **4** | **5** | **6** | **7** | **8** | **9** |
| --- | --- | --- | --- | --- | --- | --- | --- | --- | --- |
| **1** | - | 1.70 | 1.99 | 0.00 | 0.00 | 0.00 | 0.00 | 0.00 | 0.00 |
| **2** | 4.58 | - | 2.41 | 0.00 | 0.00 | 0.00 | 3.01 | 0.00 | 2.37 |
| **3** | 0.00 | 0.66 | - | 0.00 | 0.00 | 3.24 | 0.73 | 0.00 | 1.23 |
| **4** | 3.43 | 0.00 | 3.59 | - | 0.00 | 2.06 | 0.00 | 4.25 | 0.00 |
| **5** | 3.84 | 0.00 | 1.66 | 0.00 | - | 0.00 | 2.98 | 1.77 | 0.00 |
| **6** | 0.00 | 0.00 | 2.29 | 0.00 | 1.31 | - | 3.62 | 0.00 | 2.17 |
| **7** | 0.00 | 0.66 | 1.70 | 0.00 | 0.00 | 0.00 | - | 6.30 | 0.00 |
| **8** | 0.00 | 2.77 | 0.00 | 2.11 | 0.00 | 5.21 | 5.39 | - | 2.95 |
| **9** | 0.00 | 0.00 | 1.45 | 0.00 | 3.86 | 4.81 | 0.00 | 0.00 | - |

Note. Edge weights from the cross-sectional network of Wave 1 are shown above the diagonal, and edge weights from the cross-sectional network of Wave 2 are shown below the diagonal (*N*=335). Node abbreviations: 1 – Betting more than one can afford, 2 – Tolerance, 3 – Chasing losses, 4 – Borrowing money, 5 – Recognizes one has a problem, 6 – Health problems, 7 – Criticized by others, 8 – Financial problems, 9 – Feelings of guilt.


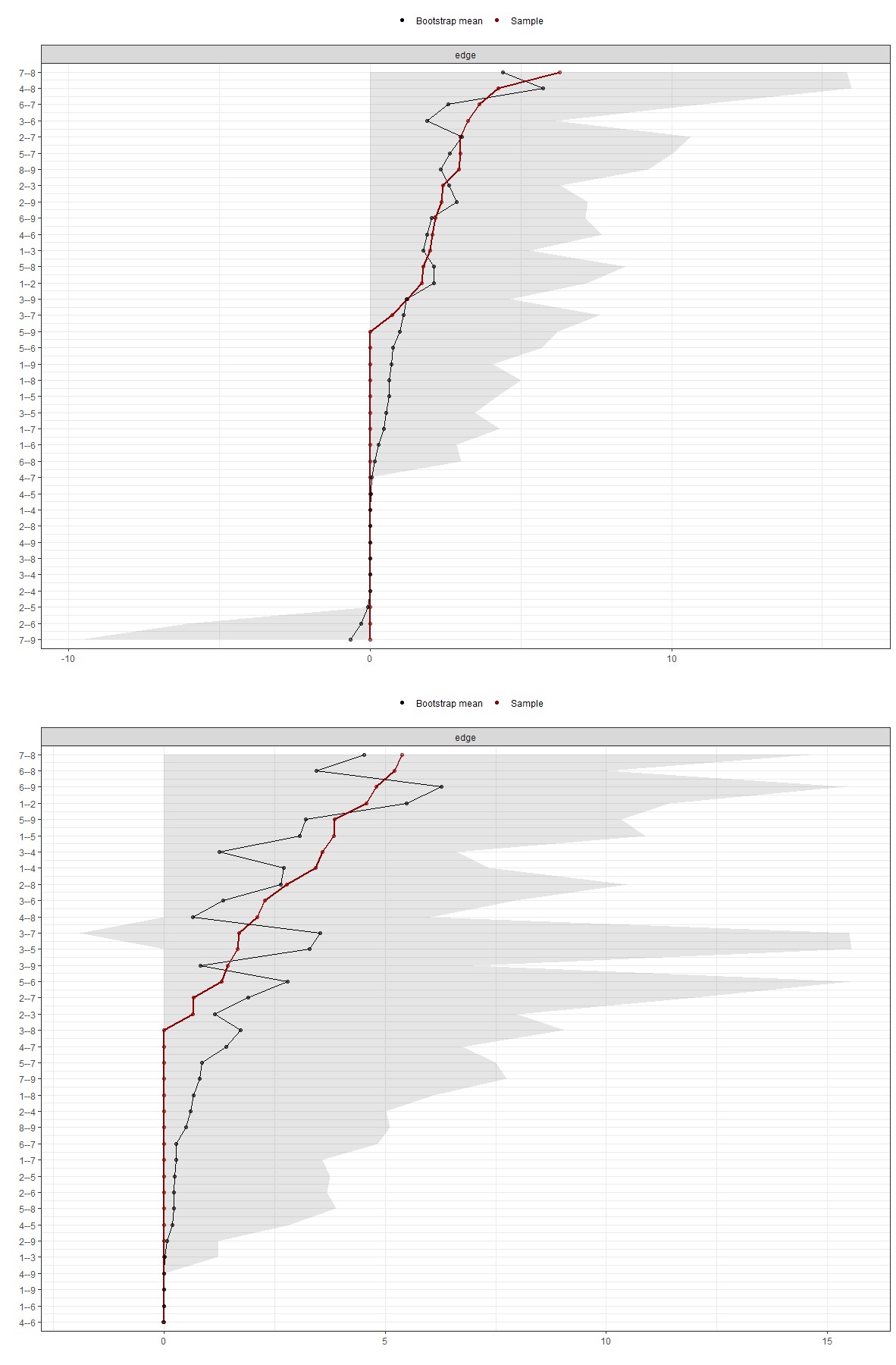


Supplementary Figure 1. Accuracy of edges weights of the cross-sectional networks of problem gambling symptoms from Wave 1 (upper figure) and Wave 2 (lower figure). Red dots and lines are edge weights from the samples. Black dots and lines are edge weights that are generated based on 1000 random bootstrap samples, and greyed areas are the bootstrap-generated confidence intervals for each edge weight. Node abbreviations: 1 – Betting more than one can afford, 2 – Tolerance, 3 – Chasing losses, 4 – Borrowing money, 5 – Recognizes one has a problem, 6 – Health problems, 7 – Criticized by others, 8 – Financial problems, 9 – Feelings of guilt.


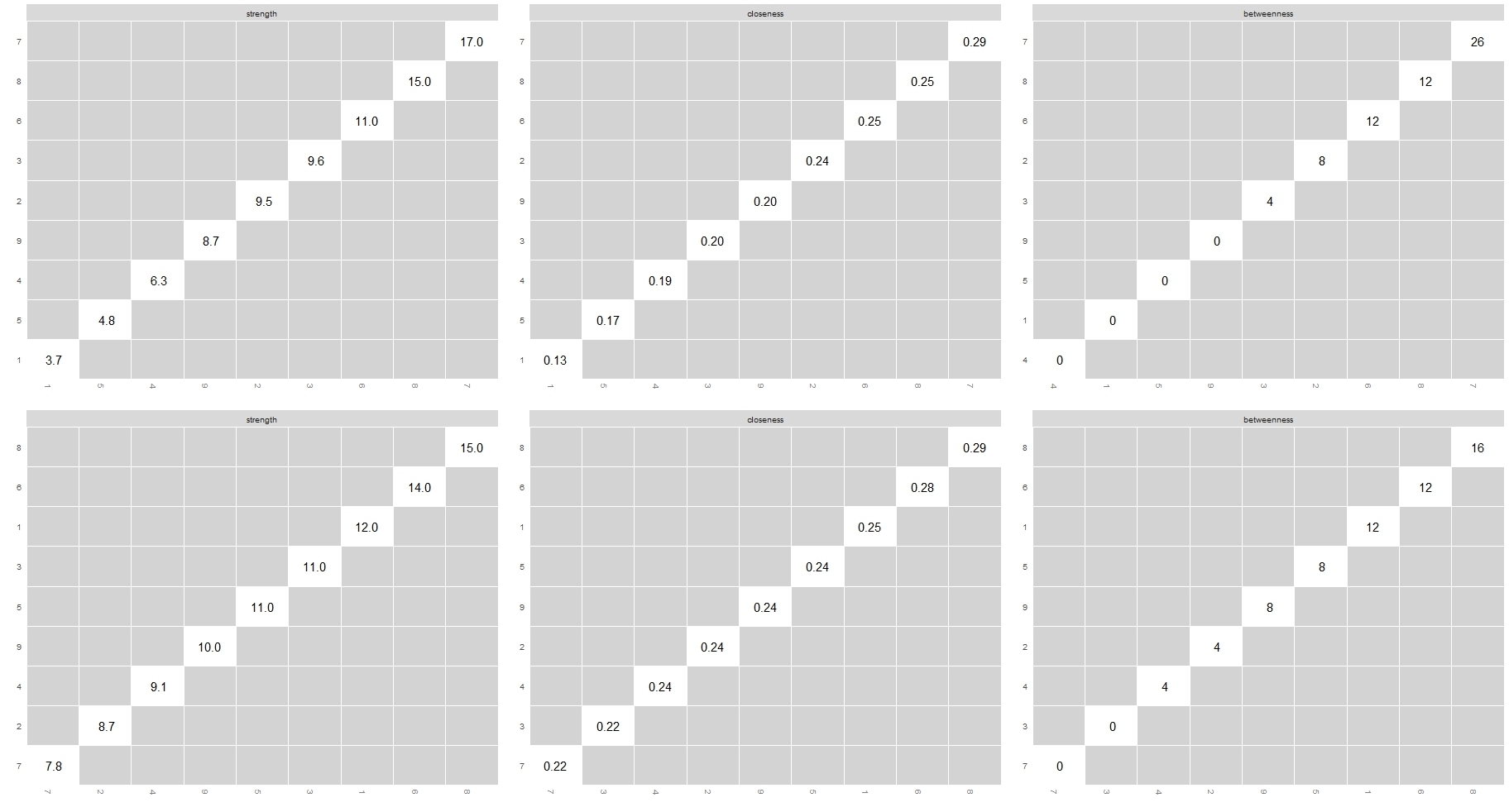


Supplementary Figure 2. Bootstrapped difference tests for centrality indices related to cross-sectional networks of problem gambling symptoms from Wave 1 (upper row) and Wave 2 (lower row). Grey boxes indicate non-significant differences (*p*>.050). Values in the diagonal are the raw centrality estimates for the given node. Node abbreviations: 1 – Betting more than one can afford, 2 – Tolerance, 3 – Chasing losses, 4 – Borrowing money, 5 – Recognizes one has a problem, 6 – Health problems, 7 – Criticized by others, 8 – Financial problems, 9 – Feelings of guilt.


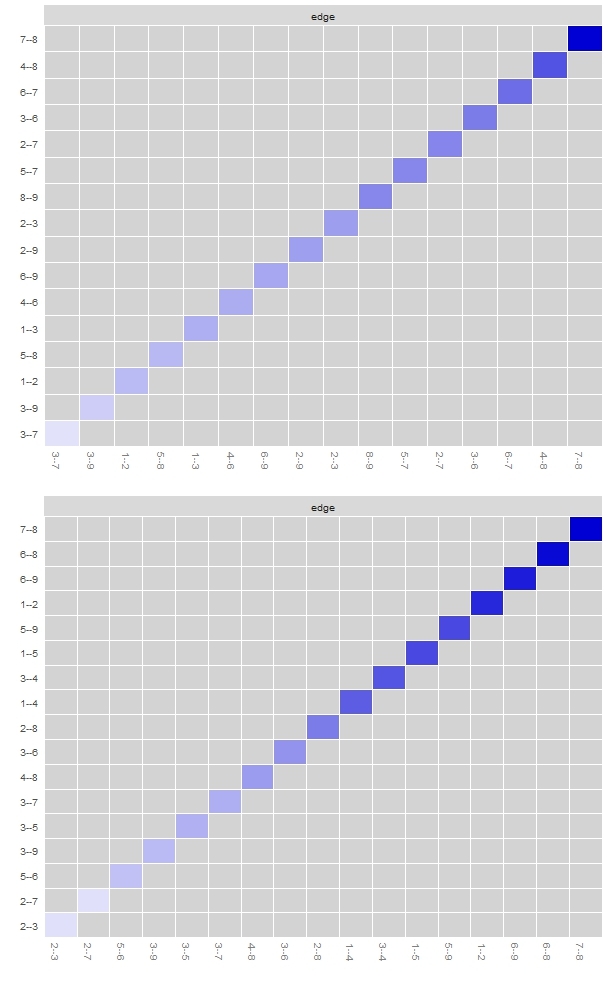


Supplementary Figure 3. Bootstrapped difference tests between edge weights related to cross-sectional networks of problem gambling symptoms from Wave 1 (upper figure) and Wave 2 (lower figure). Non-significant and significant (*p*<.050) differences between edges are indicated by grey and black boxes, respectively. Colored boxes in the diagonal indicate the strength of relationships. Node abbreviations: 1 – Betting more than one can afford, 2 – Tolerance, 3 – Chasing losses, 4 – Borrowing money, 5 – Recognizes one has a problem, 6 – Health problems, 7 – Criticized by others, 8 – Financial problems, 9 – Feelings of guilt.


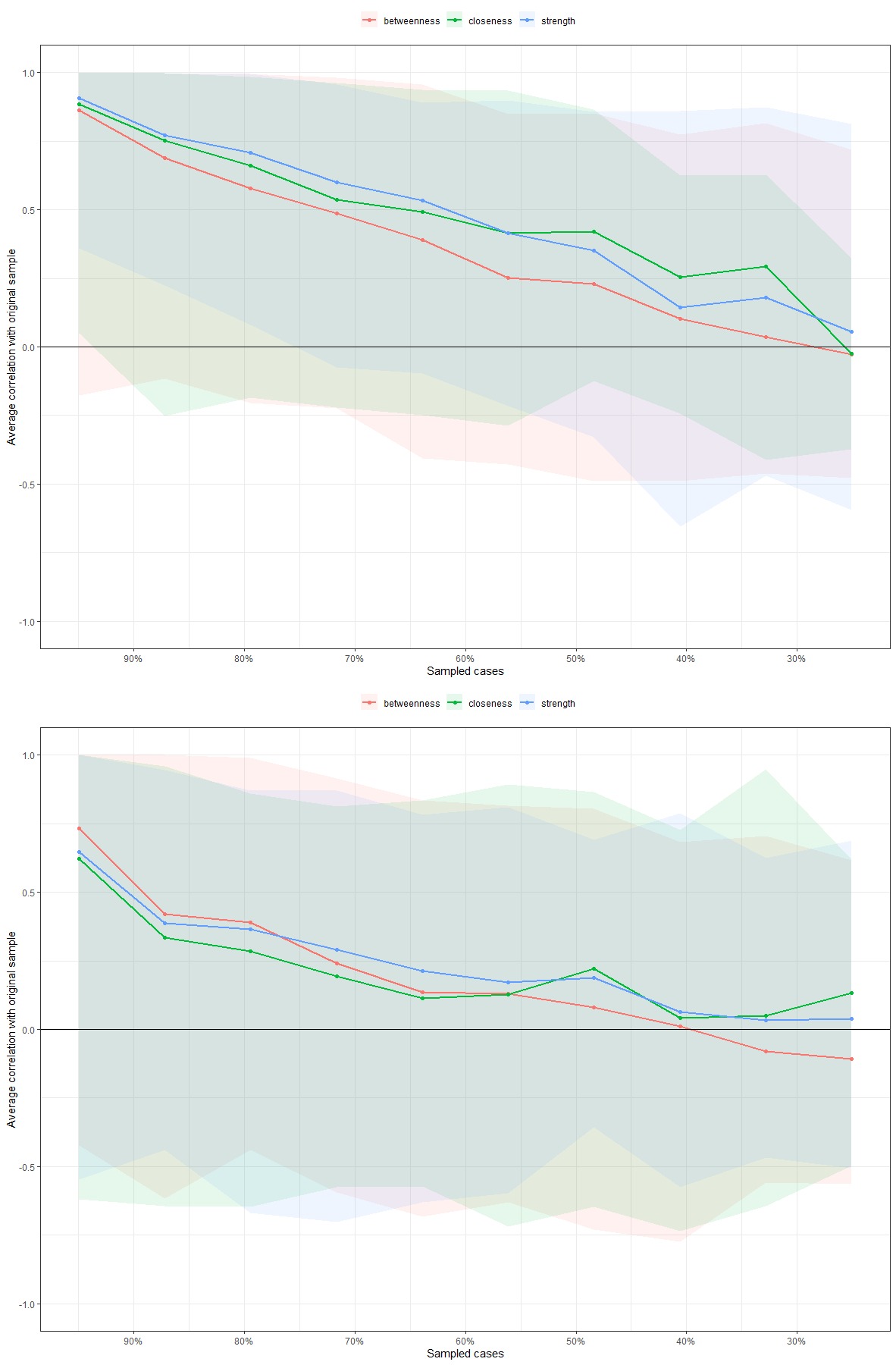


Supplementary Figure 4. Correlation stability of centrality indices related to cross-sectional networks of problem gambling symptoms from Wave 1 (upper figure) and Wave 2 (lower figure). Solid lines link the means of correlations from subsets with increasing number of excluded participants. Colored areas show the range of correlations between 2.5th quantile and 97.5th quantile.

Supplementary Table 2. Cross-lagged LASSO regression predictive effects of problem gambling symptoms between Waves 1 and 2

|  | | **Outcome variables: symptoms at Wave 2** | | | | | | | | |
| --- | --- | --- | --- | --- | --- | --- | --- | --- | --- | --- |
|  |  | **1** | **2** | **3** | **4** | **5** | **6** | **7** | **8** | **9** |
| **Predictor variables: symptoms at Wave 1** | **1** | 0.00 | 0.00 | 0.00 | 0.00 | 0.00 | 0.00 | 0.00 | 0.00 | 0.00 |
|  | **2** | 0.00 | 0.00 | 0.00 | 0.00 | 0.38 | 0.00 | 0.69 | 0.06 | 0.00 |
|  | **3** | 0.00 | 0.00 | 0.00 | 0.00 | 0.00 | 0.00 | 0.00 | 0.00 | 0.00 |
|  | **4** | 0.00 | 0.00 | 0.00 | 0.00 | 0.00 | 0.00 | 0.00 | 0.00 | 0.00 |
|  | **5** | 0.00 | 0.00 | 0.00 | 0.00 | 0.00 | 0.00 | 0.00 | 0.00 | 0.00 |
|  | **6** | 1.15 | 0.00 | 0.94 | 0.00 | 1.65 | 0.94 | 1.51 | 1.45 | 0.00 |
|  | **7** | 0.00 | 0.00 | 0.00 | 0.00 | 0.00 | 0.00 | 0.00 | 0.00 | 0.00 |
|  | **8** | 0.00 | 0.00 | 0.00 | 0.00 | 0.00 | 0.00 | 0.00 | 0.00 | 0.00 |
|  | **9** | 0.00 | 0.00 | 0.00 | 0.00 | 0.00 | 0.00 | 0.00 | 0.00 | 0.00 |

Note. Values between the problem gambling symptoms are unstandardized regression coefficients (B) based on binary logistic regression models. Node abbreviations: 1 – Betting more than one can afford, 2 – Tolerance, 3 – Chasing losses, 4 – Borrowing money, 5 – Recognizes one has a problem, 6 – Health problems, 7 – Criticized by others, 8 – Financial problems, 9 – Feelings of guilt.


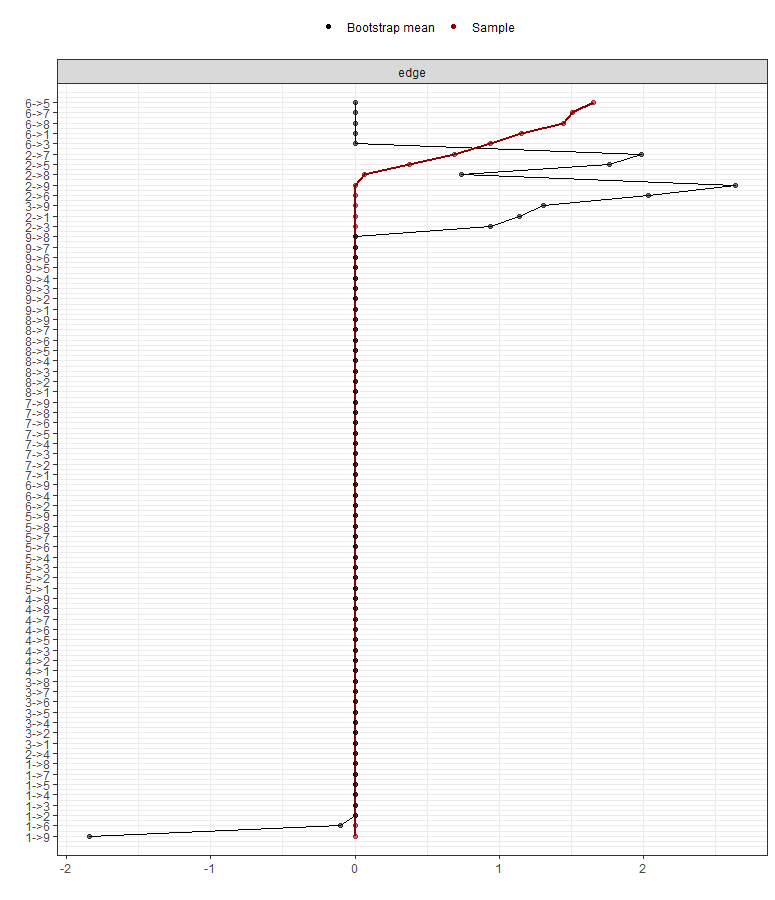


Supplementary Figure 5. Accuracy of edges weights of the cross-lagged network of problem gambling symptoms. Red dots and lines are edge weights from the samples. Black dots and lines are edge weights that are generated based on 1000 random bootstrap samples. Node abbreviations: 1 – Betting more than one can afford, 2 – Tolerance, 3 – Chasing losses, 4 – Borrowing money, 5 – Recognizes one has a problem, 6 – Health problems, 7 – Criticized by others, 8 – Financial problems, 9 – Feelings of guilt.


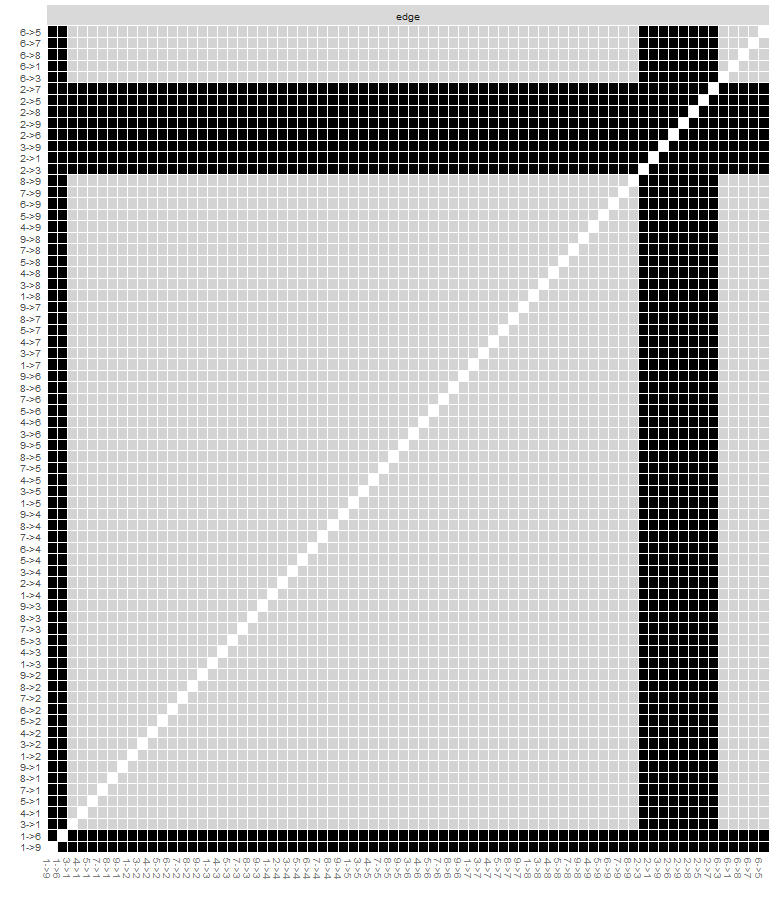
 Supplementary Figure 6. Bootstrapped difference tests between edge weights of the cross-lagged network of problem gambling symptoms. Non-significant and significant (*p*<.050) differences between edges are indicated by grey and black boxes, respectively. Node abbreviations: 1 – Betting more than one can afford, 2 – Tolerance, 3 – Chasing losses, 4 – Borrowing money, 5 – Recognizes one has a problem, 6 – Health problems, 7 – Criticized by others, 8 – Financial problems, 9 – Feelings of guilt.


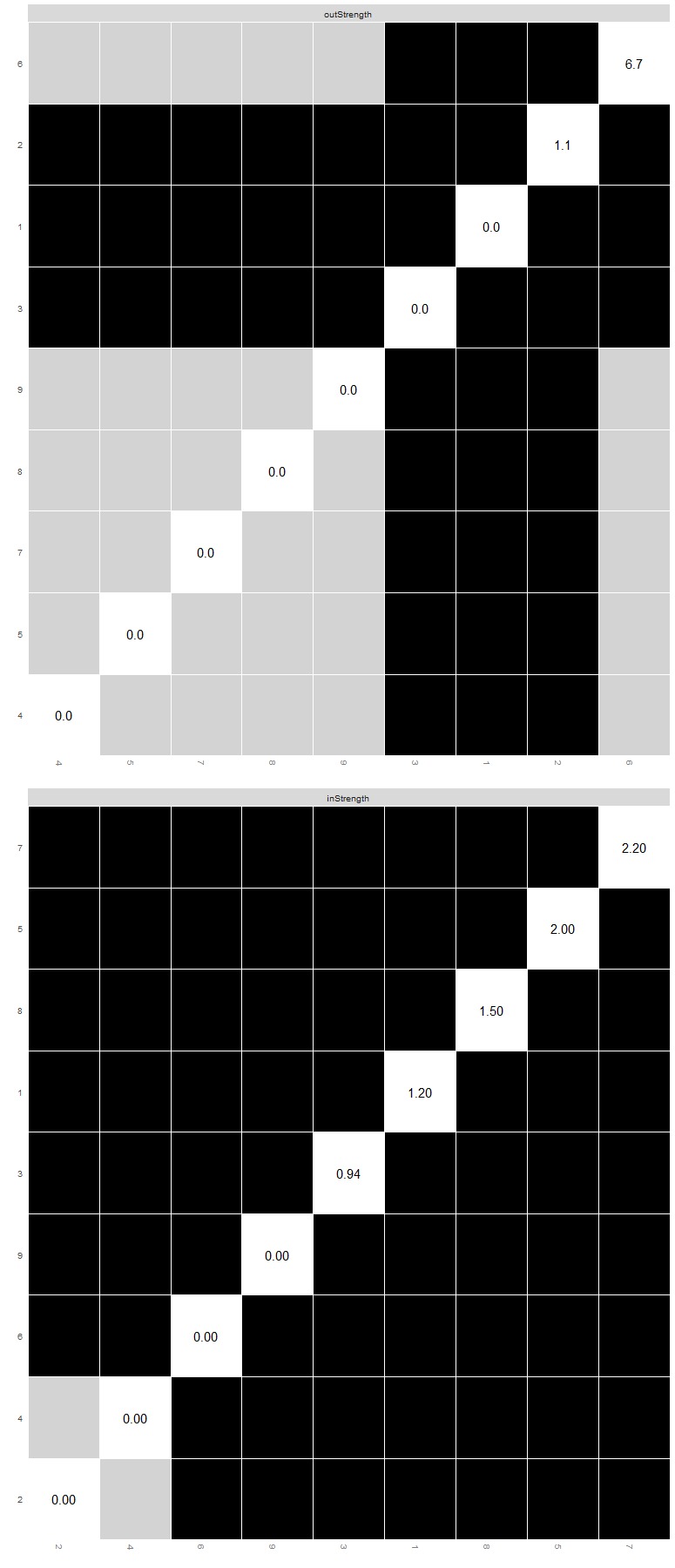


Supplementary Figure 7. Bootstrapped difference tests for centrality indices related to the cross-lagged network of problem gambling symptoms. Non-significant and significant (*p*<.050) differences between edges are indicated by grey and black boxes, respectively. Values in the diagonal are the raw centrality estimates for the given node. Node abbreviations: 1 – Betting more than one can afford, 2 – Tolerance, 3 – Chasing losses, 4 – Borrowing money, 5 – Recognizes one has a problem, 6 – Health problems, 7 – Criticized by others, 8 – Financial problems, 9 – Feelings of guilt.
